# A randomised controlled trial of total hip arthroplasty versus resurfacing arthroplasty in the treatment of young patients with arthritis of the hip joint

**DOI:** 10.1186/1471-2474-11-8

**Published:** 2010-01-14

**Authors:** Juul Achten, Nick R Parsons, Richard P Edlin, Damian R Griffin, Matthew L Costa

**Affiliations:** 1 Clinical Sciences Research Institute, Warwick University, Clifford bridge road, Coventry, CV2 2DX, UK; 2 Leeds institute of health sciences, Leeds University, Worsley Building, Leeds, LS2 9JT, UK

## Abstract

**Background:**

Hip replacement (arthroplasty) surgery is a highly successful treatment for patients with severe symptomatic arthritis of the hip joint. For older patients, several designs of Total Hip Arthroplasty have shown excellent results in terms of both function and value for money. However, in younger more active patients, there is approximately a 50% failure rate at 25 years for traditional implants. Hip resurfacing is a relatively new arthroplasty technique. In a recent review of the literature on resurfacing arthroplasty it was concluded that the short-term functional results appear promising but some potential early disadvantages were identified, including the risk of femoral neck fracture and collapse of the head of the femur. The aim of the current study is to assess whether there is a difference in functional hip scores at one year post-operation between Total Hip Arthroplasty and Resurfacing Arthroplasty. Secondary aims include assessment of complication rates for both procedures as well cost effectiveness.

**Methods/design:**

All patients medically fit for surgery and deemed suitable for a resurfacing arthroplasty are eligible to take part in this study. A randomisation sequence will be produced and administered independently. After consenting, all patients will be clinically reviewed and hip function, quality of life and physical activity level will be assessed through questionnaires. The allocated surgery will then be performed with the preferred technique of the surgeon. Six weeks post-operation hip function will be assessed and complications recorded. Three, six and 12 months post-operation hip function, quality of life and physical activity level will be assessed. Additional information about patients' out-of-pocket expenses will also be collected.

**Trial registration:**

Current Controlled Trials ISRCTN33354155

UKCLRN portfolio ID 4093

## Background

Hip arthroplasty is a procedure which has been performed by orthopaedic surgeons for decades, with improvements to the procedure and the implants being made almost continuously over that time. For older patients, several designs of Total Hip Arthroplasty (THA) have shown excellent results in terms of both function and value for money [1]. However, in younger more active patients, there is an approximate 50% failure rate at 25 years for traditional implants [2]. Modern THA designs with hard bearing surfaces may improve upon these results [3], but long-term evidence is lacking. The early results of Resurfacing Arthroplasty (RSA), a new technique where the proximal femoral anatomy is preserved, suggest a 98% survival at five years [4]; which is as good as any of the existing THA's [1]. However, no long-term data exists. In a recent review of the literature on RSA it was concluded that although the short-term functional results appear promising, some potential early disadvantages were identified; including risk of femoral neck fracture, avascular necrosis of the head of the femur and acetabular bone stock sacrifice [5].

At present, only a few randomised trials have been performed comparing the traditional techniques of THA with the resurfacing technique. These studies have focused on the technical aspects of the procedure, such as the position of the implants or the amount of bone removed during the resurfacing procedure [6,7]. There are currently no published results of randomised trials comparing these two different methods of hip arthroplasty using hip function, activity level and patient quality of life as the primary outcome measure. In a non-randomised study, Pollard et al. [8] reported no difference in the Oxford hip score or the rate of revision between RSA and THA, however patients in the RSA group where shown to have higher activity levels after the procedure and were more likely to be involved in activities such as running and heavy manual labour. Furthermore, quality of life scores were found to be higher in the RSA group compared to the THA group. In another recent non-randomised study similar results were reported, with higher activity scores and range of movement scores for the resurfacing group [9]. In this study complication rates and reoperation rates at 2 year follow-up were the same for the two techniques, however the study design was poor as the two treatment groups were not comparable with regards to sex, age and pre-operative functional scores. To provide surgeons and patients with accurate information regarding early function and complication rates, we feel that it is essential to perform a randomised trial comparing THA with RSA.

The null hypothesis for this trial is that there is no difference in functional hip scores (Harris and Oxford Hip score) at one year post-operation between Total Hip Arthroplasty and Resurfacing Arthroplasty.

## Methods/design

### Design

This is a single-blind randomised controlled trial. This study has been reviewed by the Coventry Research Ethics Committee under reference number 07/Q2802/26. The study was approved on the 9^th ^of May 2007. The research carried out is in compliance with the Helsinki Declaration.

### Study participants

#### Inclusion criteria

The only eligibility criterion for participation in this study is that the patient is medically fit for an operation and suitable for a RSA (all patients that are suitable for RSA are also suitable for a THA). These broad eligibility criteria should ensure that the results of the study can readily be generalised to the wider population.

#### Exclusion criteria

Contra-indications to surgery, defined as: (i) severe cardiac impairment, e.g. heart or valve replacement, arrhythmia, previous myocardial infarction, (ii) severe respiratory impairment, e.g. chronic obstructive pulmonary disease, asthma that has required hospital admission, or (iii) any other systemic medical condition that would produce a specific contraindication to a general anaesthetic.

Evidence that the patient would be unable to adhere to trial procedures or complete questionnaires, such as dementia or intravenous drug abuse

If a recruited patient requires a contra-lateral hip replacement during the trial period, this second hip cannot be included in the study (i.e cannot be randomised) since the result of this intervention would not be independent from the first intervention.

#### Post randomisation withdrawals

Participants may withdraw from the trial at any time without prejudice. If patients decide to have the treatment to which they were not randomised, participants will be followed-up wherever possible and data collected as per the protocol until the end of the trial. The primary analysis will be on an intention-to-treat basis with a secondary per-protocol analysis. If patients decide to have neither treatment, they will be withdrawn from the trial and not included in the analysis.

The two treatments in this study are THA and RSA. Each patient will undergo the allocated surgery according to the preferred technique of the operating surgeon. The surgeons involved in the study use a variety of surgical approaches to the hip joint: anterolateral, posterior and, for the resurfacing patients, the Ganz approach. Some surgeons use an uncemented THA for younger more active patients whilst others prefer a more traditional cemented design or a 'hybrid' of the two. In the same way, some surgeons cement the femoral component of a RSA while others prefer an uncemented component.

In summary, the details of the surgery will be left entirely to the discretion of the supervising surgical consultant to ensure that the results of the trial can be generalised to as wide a group of patients as possible.

### The Study intervention

#### Total Hip Arthroplasty

In a THA, the femoral head is removed along with the majority of the femoral neck. The femoral shaft is 'reamed' to open up the femoral canal. The femoral component is then inserted into the canal and the articulating femoral head is placed onto the neck of the femoral component.

#### Resurfacing Arthroplasty

In a RSA, the articular surfaces of the femoral head are removed but the neck is left in-situ. The femoral component is then impacted onto the patient's own femoral neck.

**Figure 1:**
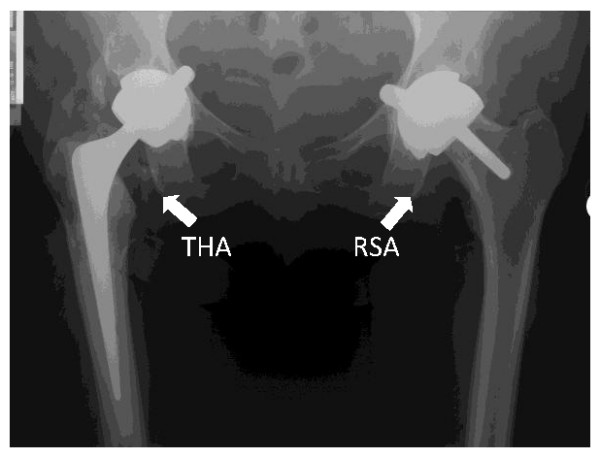
**X-ray showing a Total Hip Arthroplasty (THA) and a Resurfacing Arthroplasty (RSA)**.

In both forms of Arthroplasty, the acetabulum is reamed and the acetabular component inserted into the 'socket'. The radiograph (Figure 1) shows a THA on the right side and a RSA on the left side.

#### Rehabilitation

Standardised rehabilitation plans will be implemented for all of the patients; as outlined in the University Hospitals Coventry and Warwickshire NHS Trust 'Hip Replacement: A Guide for Patients' booklet. This includes information on early exercises, precautions to be followed for the first three months, functional activity and later stage exercises. A photocopy of the 'joint rehabilitation discharge letter' will be made as proof that all of the patients completed these tasks to a standard level before discharge.

#### Follow-up

Patients will continue in routine clinical follow-up, as per their surgeon's practice. For this trial, the primary outcome point will be at one year, with other planned assessments at six weeks, 3 months and six months after operation. At all these occasions patients will come to the clinic to be reviewed by the research associate and if necessary a doctor.

### Study objectives

There are three main objectives of this randomised controlled trial:

1. To quantify and draw inferences on observed differences in primary and secondary outcomes measures between the trial treatment groups at one year post-operatively.

2. To detemine the complication rate of Resurfacing Hip Arthroplasty versus Total Hip Arthroplasty at one year post-operatively.

3. To investigate, using appropriate statistical and economic analysis methods, the resource use, and thereby the cost effectiveness, of Resurfacing Hip Arthroplasty versus Total Hip Arthroplasty.

### Outcome measures

This study has two primary outcome measures; the ***Oxford Hip Score ***[10] and the ***Harris Hip Score ***[11]. The Oxford Hip Score is a validated questionnaire which is self-administered. It consists of 12 items related to daily tasks directly influenced by poor hip function. The Harris Hip score is also a hip function questionnaire which includes items reflecting a patient's ability to perform normal daily activities but also contains objective measurements such as range-of-movement.

Three secondary outcome measures will be used in this trial; (i) ***EuroQol 5D (EQ-5D)***: A validated quality of life questionnaire consisting of 5 questions related to daily activities scored on a 3-point ordinal score scale. Answers can be combined using published algorithms to obtain health-related quality of life values (ii) ***Disability Rating Index***, a self-administered, 12-item VAS questionnaire assessing the patient's own rating of disability and (iii) ***Paffenbarger Physical Activity Questionnaire***, this questionnaire assesses the number of calories a patient expends on leisure and physical activities built on an assessment of energy spent on average week and weekend days, as well as additional energy spent during sporting activities throughout the year. All complications will be recorded during the course of the trial.

Resource use will be monitored for the economic analysis. NHS costs associated with each arm of the trial will be estimated using data from both national sources (NHS reference costs, PSSRU reference costs [12]) and the finance departments of the hospitals and services concerned. The cost consequences following discharge, including NHS costs and patients' out-of-pocket expenses will be recorded via a short questionnaire which will be administered at 3, 6 and 12 months post surgery. Patient self-reported information on service use has been shown to be accurate in terms of intensity of use of different services [13].

In this study, we will use techniques common in long term cohort studies to ensure minimum loss at recruitment, such as collection of multiple contact addresses and telephone numbers, mobile telephone numbers and email addresses. Every possible effort will be made to minimise loss to follow-up, with a target of complete follow-up. Using these techniques, we are confident that loss will not exceed 10%. In the event of a participant being lost to the final follow-up at one year, we will consider, on the advice of the trial statistician, imputing missing primary outcome data from interim scores.

A system of reminders will be instituted to ensure that return to clinic at three, six and twelve months is as complete as possible. The Research Associate will phone participants to make an appointment, after two weeks non-response a letter will be send out to the patient. The letter will be followed up by phone call after 1-2 weeks. If there is no response from the participant, they will be classed as a 'non-responder' and the case closed.

### Sample size

Previous work has shown the Oxford Hip Score (OHS) and the Harris Hip Score (HHS) to be reliable measures of hip function after surgery, as measured by Cronbach's alpha statistic for internal consistency [10,14]. Correlation analysis has indicated that both measures are reproducible, i.e. their test-retest reliability is good, and previous studies [15] have shown them to be strongly correlated, indicating that both scoring systems are measuring the same latent characteristic (hip function). However, the self-administered OHS has achieved much higher follow-up rates than the more widely used HHS [15]. Therefore, in order to provide the best assessment of patient experience of hip function after surgery and also to allow dissemination of the results of this trial to as wide a potential audience as possible in resulting publications, we prudently choose to use both the OHS and the HHS as primary outcome measures.

Harris Hip Score. There is less available specific information on previously observed variability for the HHS, than for the OHS. Assuming Normally distributed scores, the required numbers of patients in each arm of the trial are shown in Table 1, based on an independent samples t-test (at the 5% level) for assumed standard deviations (SD) of 13 and 15 [15] and minimum clinically important differences (MCIDs) of 7 and 10 [16](PS, power and sample size software, available at http://biostat.mc.vanderbilt.edu/wiki/Main/PowerSampleSize

**Table 1:** Patient number required for MCID in Harris Hip Score at different power levels.

	MCID = 7		MCID = 10	
**Power**	**80%**	**90%**	**80%**	**90%**

SD				

13	**55**	**73**	28	37

15	73	97	36	48

MCID: Minimum clinically important difference

The minimum clinically important differences used for this sample size calculation give effect sizes of around 0.6; a medium to large effect size using the conventional descriptions of these terms [17]. More reliable data for determination of the MCID is available for the OHS.

Oxford Hip Score. The best estimate for the standard deviation for the OHS is 9 and the MCID is between 5 and 7 points on the OHS scale, calculated from a previous comprehensively reported study [18]. Using these estimates the required numbers of patients in each arm of the study, for a two-sample t-test (at the 5% level) are shown in Table 2.

**Table 2:** Patient number required for minimum MCID in Oxford Hip Score at different power levels.

	MCID = 5		MCID = 7	
**Power**	**80%**	**90%**	**80%**	**90%**

Sample size	**52**	**69**	27	36

MCID: Minimum clinically important difference

The results for the OHS and the HHS at the 5% significance level, based on the best available estimates of MCID (5 and 7 score points respectively) are very similar. Both scores are intended to measure patient hip function, so it is not unexpected that the sample sizes obtained using these scores give very similar results. An adjustment to the significance level for multiple comparisons resulted in only a small increase (2-5 patients) in the required numbers.

In summary, this study will use two primary outcome measures the Oxford Hip Score and the Harris Hip Score. The aim will be to recruit 78 patients in each group as this will provide sufficient participants to obtain a power of 90% for both primary outcome measures. With an allowance for 10% drop-out, the total number of patients required will be **172**. If recruitment proves to be problematic during the course of the trial, then with the agreement of the trial steering committee the target will be lowered and the more usual 80% power level will be considered sufficient. For this scenario, the total number of patients required will be 120 (including 10% for drop-out).

### Randomisation

A randomisation sequence has been independently generated on a computer. The sequence is stratified by supervising consultant orthopaedic surgeon. The randomisation sequence will be held by the Clinical Trials Unit of the University of Warwick. After patients have consented to take part in the trial, pre-operation outcome scores will be collected. Thereafter, the randomisation officer will be alerted by telephone of a new enrolment. The randomisation officer will provide the surgeons secretary with the treatment allocation for the patient which will then be entered onto the hospital system.

### Blinding

Before this trial was designed, patients undergoing resurfacing arthroplasty of the hip in our department were given a different pre-operative information sheet from those having a total hip arthroplasty; this reflected the existing evidence regarding the different risk/benefit profile of the two procedures. Therefore, we do not consider it ethical to blind the patients to their treatment allocation within the trial. The patients will be informed, by letter, of their treatment allocation in the week after they have given their consent. However, we will collect pre-operative information about the patients' preference for one or other treatment.

The consultants will, of course, not be blind to the treatment but will take no part in the post-operative assessment of the patients.

The research associate who performs the pre- and post- treatment outcome measurements will be blind to treatment allocation throughout the entire study. Patients will be asked not to discuss their allocated treatment with the research associate.

### Statistical analysis

The main analysis will investigate differences in the primary outcome measures, the Harris Hip Score and the patient-reported Oxford Hip Score, between the two treatment groups on an intention-to-treat basis. The differences between treatment groups will be assessed using an independent samples t-test at 12 months post-operatively at the 5% level. Test levels, for the two primary outcome measures will be adjusted using the methods of Holm-Bonferroni [19] to allow for the multiple comparisons. A linear regression analysis will also be used to quantify the effects of the treatment groups on each of the primary outcome measures, after adjusting for the effects of a range of other important, potentially confounding, factors (e.g. age, gender) recorded for each patient. Subsidiary analyses will report results of tests for secondary outcome measures and primary outcome measures at interim occasions. Interim analyses will be performed only where directed by the Data Monitoring Committee.

The second main statistical objective of the trial is to determine, and compare, the complication rate of RSA and THA at one year post-operatively. All complications will be recorded during the trial, and rates will be determined at 12 months post-operatively and compared between treatment groups using a chi-squared (at the 5% level).

### Economic analysis

The economic evaluation will estimate the incremental cost effectiveness of RSA compared to THA from the perspective of the UK NHS. A within trial (12 month) analysis will be conducted, with lifetime results extrapolated from trial data, registry data, and the literature using a patient level simulation model. These analyses will have common features: both will use a quality-adjusted life-year (QALY) outcome measure based on EQ-5D data and the 10 year MVH TTO tariff [20]; both will use a payer (NHS and Social Services) perspective for the main case analysis; costs will be obtained from resource using national databases (NHS Reference Costs, the BNF, and PSSRU Costs of Health and Social Care) and local data sources (UHCW finance department).

The within trial analysis will consider cost-effectiveness within the first 12 months of treatment. This analysis will be based on quality of life data collected at discharge (baseline) and at 3, 6, and 12 months post operatively, with QALYs obtained by calculating the area under the curve. Resource usage will be estimated from trial forms plus patient questionnaires detailing other NHS contacts and personal expenditures within the trial period, with costs assessed as above.

As hip replacement and failure of hip replacements may have an impact upon mortality as well as morbidity, it will be necessary to adopt a lifetime time horizon to estimate the full incremental value of RSA compared to THA. Therefore we will construct a patient-level decision analytic cost effectiveness model to estimate the expected incremental cost per life year gained for RSA compared to THA. Whilst the trial dataset will be used to provide parameters for several states, the main data sources for the extrapolation will come from existing literature and registries as the model horizon greatly exceeds the trial length. The model will comprise states specific to both RSA and THA (surgery, recovery, success and failure), with adjustments for THA revision surgeries (as feasible). Quality of life figures will be obtained from a combination of trial data and literature/registries. Mortality will be represented using age/gender specific rates, with additional mortality assigned in surgical, recovery and failure states (as feasible). Failure rates for devices will be based on survival analysis, with the type and complexity of relationships determined by Bayesian decision criteria. Costs will be obtained from the within-trial analysis by status and used to inform state-specific costs, with existing literature and expert judgement used where necessary. The model will use a standard discount rate of 3.5 and parameter uncertainty will be addressed through probabilistic sensitivity analysis, with any inflation-adjustments based on the HCHS Pay and Prices Index. Wherever possible, the results of all analyses will be presented in a simple, easy to follow manner using standard techniques within economic evaluation. Outputs of each analysis will be presented as Cost Effectiveness Acceptability Frontiers and Expected Net Benefit figures assuming that the cost-effectiveness threshold lies at £30,000 per QALY.

## Abbreviations

THA: Total Hip Arthroplasty; RSA: Resurfacing Arthroplasty; QALY: Quality adjusted life year; OHS: Oxford Hip Score; HHS: Harris Hip Score.

## Competing interests

The authors declare that they have no competing interests. The department of trauma and orthopaedics at UHCW receives funding from manufacturers of both total hip arthroplasty and resurfacing arthroplasty for routine post-market surveillance of implants. However, no funding has been received in relation to this trial.

## Authors' contributions

JA developed the protocol, assisted in securing grant funding and manages the running of the trial. NP developed the protocol, assisted in securing grant funding and is responsible for the statistical analysis of the trial. RE has been responsible for developing the health economics analysis part of the protocol. DG developed the protocol and is responsible for the recruitment of trial patients. MC developed the protocol, secured the grant funding, is responsible for the recruitment of trial patients, management of trial and has overall clinical responsibility for the conduct of the trial. All authors read and approved the final manuscript.

## Pre-publication history

The pre-publication history for this paper can be accessed here:

http://www.biomedcentral.com/1471-2474/11/8/prepub
